# AC Characteristics of van der Waals Bipolar Junction Transistors Using an MoS_2_/WSe_2_/MoS_2_ Heterostructure

**DOI:** 10.3390/nano14100851

**Published:** 2024-05-14

**Authors:** Zezhang Yan, Ningsheng Xu, Shaozhi Deng

**Affiliations:** State Key Laboratory of Optoelectronic Materials and Technologies, Guangdong Province Key Laboratory of Display Material and Technology, School of Electronics and Information Technology, Sun Yat-sen University, Guangzhou 510275, China; yanzzh3@mail2.sysu.edu.cn (Z.Y.); stsxns@mail.sysu.edu.cn (N.X.)

**Keywords:** two-dimensional material, van der Waals, vertically stacked, bipolar junction transistor, AC characteristics

## Abstract

Two-dimensional layered materials, characterized by their atomically thin thicknesses and surfaces that are free of dangling bonds, hold great promise for fabricating ultrathin, lightweight, and flexible bipolar junction transistors (BJTs). In this paper, a van der Waals (vdW) BJT was fabricated by vertically stacking MoS_2_, WSe_2_, and MoS_2_ flakes in sequence. The AC characteristics of the vdW BJT were studied for the first time, in which a maximum common emitter voltage gain of around 3.5 was observed. By investigating the time domain characteristics of the device under various operating frequencies, the frequency response of the device was summarized, which experimentally proved that the MoS_2_/WSe_2_/MoS_2_ BJT has voltage amplification capability in the 0–200 Hz region. In addition, the phase response of the device was also investigated. A phase inversion was observed in the low-frequency range. As the operating frequency increases, the relative phase between the input and output signals gradually shifts until it is in phase at frequencies exceeding 2.3 kHz. This work demonstrates the signal amplification applications of the vdW BJTs for neuromorphic computing and wearable healthcare devices.

## 1. Introduction

Bipolar junction transistors (BJTs), as some of the important semiconductor devices, have attracted great attention in past decades. They contain three separately doped regions, which are defined as the collector, base, and emitter. Two-dimensional (2D) materials offer significant opportunities for the construction of high-performance BJTs owing to their unique interlayered van der Waals (vdW) bonding characteristics [1,2,3]. By employing mechanical exfoliation or chemical vapor deposition (CVD), monolayer or multilayer 2D materials, including hexagonal boron nitride (h-BN) [4,5], transition metal dichalcogenides (TMDs) [6,7,8,9], and graphene [10,11,12], can be easily obtained. These ultrathin 2D materials provide possibilities for fabricating vdW BJTs with base regions of atomic thicknesses.

In the past few years, several 2D-material-based BJTs, such as BP/MoS_2_/BP [13,14,15,16], Cu_9_S_5_/PtS_2_/WSe_2_ [17], and MoS_2_/WSe_2_/MoS_2_ [18,19,20], have been fabricated successively. The static characteristics of these devices have been extensively studied, and they show promising application potential in the fields of photodetection [21,22], gas sensing [23,24], and biosensing [25]. However, the AC characteristics of vdW BJTs fabricated from 2D materials have not been reported yet, which is crucial for determining a device’s ability to process alternating signals.

In this paper, we report on a vdW BJT that was constructed by vertically stacking MoS_2_, WSe_2_, and MoS_2_ flakes in sequence. The static and AC characteristics of the device were investigated in a common emitter configuration. A maximum voltage gain of around 3.5 was observed in the low-frequency range. As the operating frequency increased, the voltage gain gradually decreased to unity at 200 Hz, and the relative phase between input and output signals gradually changed from 180° to 0° at 2.3 kHz. This systematic investigation of vdW BJTs provides a direct understanding of the electrical behavior of such devices under alternating current conditions, which could potentially aid in the utilization of the vdW BJTs in wearable healthcare devices and future neuromorphic applications.

## 2. Materials and Methods

### 2.1. Device Fabrication

A controlled multistep dry transfer process was employed to fabricate the vdW BJT [26]. Firstly, the WSe_2_ and MoS_2_ flakes were exfoliated from the bulk crystals supplied by HQ Graphene Company (Groningen, The Netherlands). Then, using the dry transfer technique, MoS_2_, WSe_2_ and MoS_2_ sheets were stacked onto a clean 300 nm SiO_2_/Si substrate in sequence. In this case, the top and bottom MoS_2_ sheets were separated by the middle WSe_2_ flake. Thirdly, maskless lithography was utilized to define the locations of the metal electrodes and thermal evaporation was employed to deposit Cr/Ag metals with thicknesses of 10 nm and 100 nm. Finally, the device underwent a two-hour annealing process in an argon atmosphere at 300 °C to eliminate the photoresistant residues and potentially facilitate Ag diffusion into the underlying MoS_2_ flakes, thereby reducing the contact resistance [27].

### 2.2. Characterization

AFM (NTEGRA Spectra, NT-MDT, Moscow, Russia) and Raman spectroscopy (In Via Reflex, Renishaw, Wotton-under-Edge, Gloucestershire, UK) instruments were employed to characterize the height profile and composition of the vdW BJT. A semiconductor parameter analyzer (B1500A, Agilent Technologies, Santa Clara, CA, USA) was used to investigate the static characteristics of the device. The AC performance of the vdW BJT was measured using an oscilloscope (DPO 7354C, Tektronix, Portland, OR, USA) and an arbitrary waveform generator (DG4062, RIGOL, Beijing, China).

## 3. Results and Discussion

**Figure 1:**
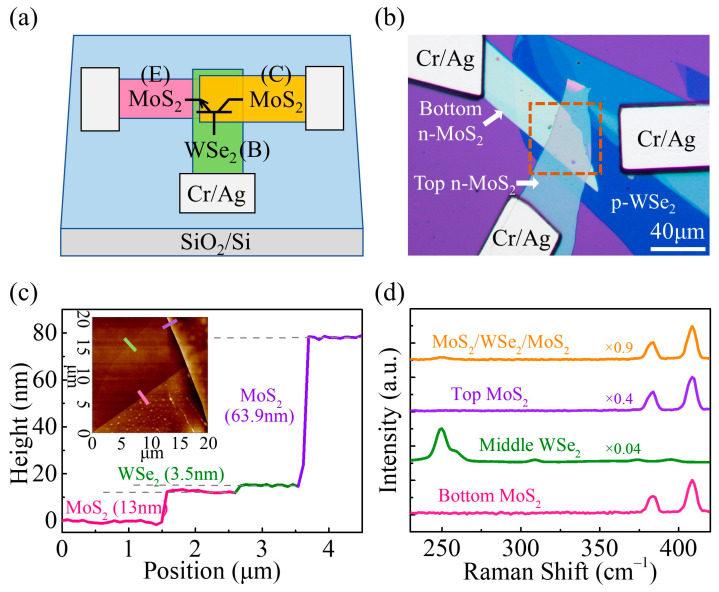
(**a**) Schematic illustration of the vdW BJT. (**b**) Optical microscope image of the vdW BJT. The inside of the dashed brown square represents the three flake overlapped region. (**c**) Height profile of the device. The dashed lines indicate the horizontal position of the steps. The inset illustrates the corresponding AFM image. (**d**) Raman spectra of the bottom MoS_2_, middle WSe_2_, top MoS_2_, and MoS_2_/WSe_2_/MoS_2_ three flake overlap regions.

Figure 1a,b show the schematic diagram and the optical image of the vertically stacked MoS_2_/WSe_2_/MoS_2_ BJT. Here, the top MoS_2_ sheet acts as the collector (C) while the bottom MoS_2_ sheet serves as the emitter (E). The multilayer WSe_2_ sheet was designed for the base (B) region. Figure 1c shows the height profile of the device. Apparently, the thicknesses of bottom MoS_2_, middle WSe_2_, and top MoS_2_are 13 nm, 3.5 nm, and 63.9 nm, respectively. To analyze the composition of the device, Raman spectra were obtained for the individual 2D materials as well as their overlap regions, as shown in Figure 1d. From the bottom MoS_2_, Raman peaks at 383.7 and 408.8 cm^−1^ can be observed. The two Raman peaks have a relatively large separation of 25.1 cm^−1^, confirming the multilayer nature of the MoS_2_ material [28,29]. The peaks at 249.8 and 258.3 cm^−1^ for the WSe_2_ flake are ascribed to the $E_{2g}^{1}$ mode and the A_1g_ mode [30]. In addition, the Raman spectra of the three flake overlap region are the sum of the Raman peaks of the MoS_2_ and WSe_2_ flakes, thereby confirming the successful fabrication of the vertically stacked heterostructure [31].

**Figure 2:**
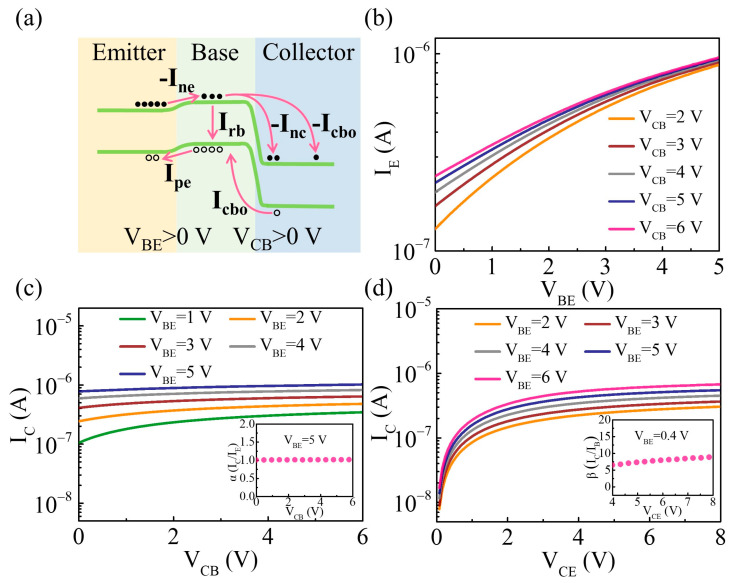
(**a**) Band diagram of the vdW BJT operating in forward-active operating mode. The orange, green, and blue areas indicate the emitter, base, and collector region of the device, respectively. (**b**) The relationship between I_E_ and V_BE_ at various fixed V_CB_ values. (**c**) The relationship between I_C_ and V_CB_ at various values of V_BE_. Inset shows α as a function of V_CB_ at a fixed V_BE_ = 5 V. (**d**) The relationship between I_C_ and V_CE_ at various fixed V_BE_ values. Inset shows β as a function of V_CE_ at a fixed V_BE_ = 0.4 V.

The static performance of the vdW BJT in common base mode was initially investigated. In this case, the base was grounded, whereas the base–collector and base–emitter junctions were separately reverse-biased and forward-biased. The band diagram of the vdW BJT operating in the forward-active operating mode is depicted in Figure 2a. Figure 2b shows the relationship between the base–emitter voltage (V_BE_) and the emitter current (I_E_) at various fixed collector–base voltages (V_CB_). With the increase in V_BE_, the depletion region of the base–emitter junction narrows, facilitating an enhanced diffusion of electrons from emitter to base. Hence, I_E_ increased with a larger V_BE_. Figure 2c illustrates the output characteristic of the vdW BJT. The V_BE_ can effectively affect the collector current (I_C_), since it can influence the electrons diffusing from the emitter. The electrons were transferred into the collector, constituting the main component of the I_C_. The common base current gain (α) was determined to be around 1.01 at V_BE_ = 5 V by calculating the ratio of I_C_ and I_E_. Figure 2d shows the output performance of the vdW BJT operating in common emitter mode. At low V_CE_ values, the collector current shows an approximately linear increase with the V_CE_, indicating the saturation region of the device [13]. Beyond the saturation region, changes in V_CE_ have minimal impact on the I_C_. Instead, the I_C_ is primarily influenced by variations in V_BE_. This region is defined as the active region of the device. A maximum current gain (β = I_C_/I_B_) of approximately 9 can be obtained at V_BE_ = 0.4 V under the common emitter configuration, as shown in Figure 2d. It is noteworthy that the MoS_2_/WSe_2_/MoS_2_ BJT has a relatively low on/off ratio, which may be attributed to the ultrathin base region. The negatively biased base–collector junction introduces an extra electric field perpendicular to the base–emitter junction, thereby diminishing the device’s on/off ratio [19,23].

**Figure 3:**
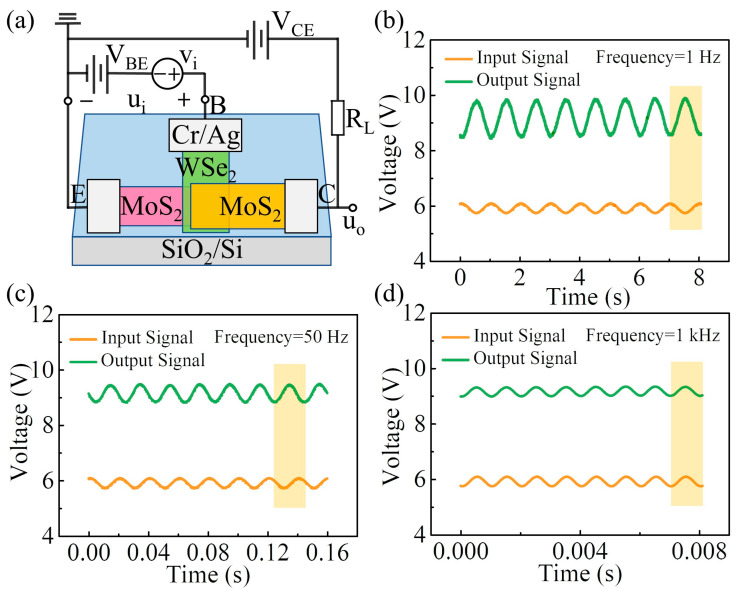
(**a**) Schematic diagram of the electric connection of the vdW BJT in common emitter mode. (**b**–**d**) Time domain characteristics of the vdW BJT operating at 1 Hz, 50 Hz, and 1 kHz, respectively. The yellow area demonstrates the relative phase between the input and output signals during one cycle of the sinusoidal signal.

Since the device showed excellent static performance, the AC characteristics of the vdW BJT operating in common emitter mode were investigated. The schematic diagram of the electrical connection is shown in Figure 3a. Here, a DC voltage (V_BE_ = 5.8 V) and a small AC voltage (v_i_) were applied to the base–emitter junction. The base–collector junction was reverse-biased by connecting the collector to another power supply (V_CE_ = 34 V) through a load resistor (R_L_ = 22 MΩ). An oscilloscope was used to monitor the input and output waveforms of the device in real time. Figure 3b illustrates the time domain characteristics of the device operating at 1 Hz. Here, an AC voltage v_i_ with an amplitude of 0.2 V is superimposed on the V_BE_, which causes the voltage applied on the base–emitter junction to fluctuate sinusoidally above and below its DC bias level. The resulting variation in I_B_ causes the output current change. Therefore, an output AC signal with an amplitude approximately 3.5 times higher than the input AC signal can be observed in the collector region.

**Figure 4:**
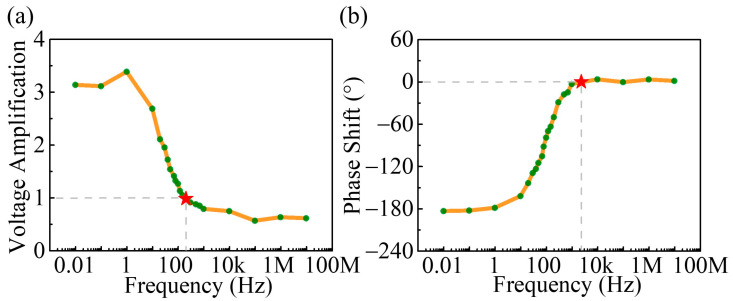
(**a**) The frequency response of the device. The red star represents the frequency corresponding to a voltage amplification of 1. (**b**) The phase response of the device. The red star represents the frequency when the input and output signals are in phase. The green dots represent experimental test results and are connected by orange lines.

The time domain characteristics of the vdW BJT operating at other frequencies were also investigated. The representative results, such as for the device operating at 50 Hz and 1 kHz, are shown in Figure 3c,d. It is worth mentioning that the voltage in the collector region was higher than that in the base region throughout the experiment, which indicates that the BJT remained in forward-active operating mode at all times. Figure 4a summarizes the common emitter voltage gain versus the operating frequency. With the increase in operating frequency, the amplitude of the output voltage signal gradually decreases until the voltage gain falls to unity at 200 Hz, indicating that the device has voltage amplification capability in the 0–200 Hz region. Figure 4b illustrates the phase response of the device. The output signal at the collector region is 180° out of phase with the input signal in the low-frequency range. As the operating frequency increases, the relative phase between the input and output signals begins to shift until the output signal is in phase with the input signal at 2.3 kHz.

Further, the frequency response of the β value can be evaluated according to the time domain characteristics of the device operating at different frequencies, as illustrated in Figure 3b–d. Here, the output current (i_C_) of the device can be determined from the output signal (u_o_) by applying the formula i_C_ = (V_CE_ − u_o_)/R_L_. As the operating frequency continuously increases, the amplitude of i_C_ is unchanged at first and then gradually decreases when the operating frequency exceeds 1 Hz. However, the input current (i_B_) is almost unaffected by the operating frequency. Therefore, the β value changing with the frequency is consistent with the trend of i_C_ changing with the frequency; that is, it decreases as the operating frequency increases. In addition, to investigate the repeatability of the AC performance of the device, several other vdW BJTs with similar configurations were fabricated and investigated. All devices exhibited similar AC characteristics. The typical test results are shown in Figure S1 of the Supplementary Materials. The performance of the 2D-material-based n-p-n BJTs reported in the literature is summarized in Table 1 [14,18,19,20,23,25].

## 4. Conclusions

In summary, a vdW BJT was fabricated by vertically stacking MoS_2_, WSe_2_, and MoS_2_ flakes in sequence. The static characteristics of the device were investigated in common emitter and common base modes, demonstrating excellent current modulation and saturation characteristics. The AC performance of the device in common emitter mode was also investigated. A phase inversion from output to input with a maximum voltage gain of around 3.5 was obtained in the low-frequency range. As the operating frequency increases, the voltage gain gradually decreases to unity at 200 Hz and the relative phase between the input and output signals gradually changes to 0° at 2.3 kHz. This work demonstrates the AC characteristics of the vdW BJT and experimentally proves the device’s ability to process alternating signals. If the issues of device array fabrication and device-to-device variation can be further addressed, this will significantly promote the application of vdW BJTs as neuromorphic devices and wearable healthcare devices.

## Figures and Tables

**Table 1:** Comparison of device performance results between this work and various previously reported 2D-material-based n-p-n BJTs [14,18,19,20,23,25].

Materials	Structure	On Current (μA)	α (DC)	β (DC)	Cutoff Frequency (Hz)	Voltage Gain	Ref.
MoS_2_/WSe_2_/MoS_2_	Vertical	0.67	1.01	9	~200	3.5	This work
MoS_2_/WSe_2_/MoS_2_	Vertical	0.001	0.97	12	/	/	[19]
MoS_2_/WSe_2_/MoS_2_	Vertical	0.054	~1	150	/	/	[18]
MoTe_2_/GeSe/MoTe_2_	Vertical	0.014	0.95	29.3	/	/	[25]
WS_2_/GeSe/WS_2_	Vertical	7.48	1.11	20.7	/	/	[23]
MoS_2_/WSe2/MoS_2_	In-plane	0.001	/	3	/	/	[20]
MoS_2_/BP/MoS_2_	Lateral	0.06	0.98	41	/	/	[14]

## Data Availability

The data presented in this article are available upon request from the corresponding author.

## Supplementary Materials

The following supporting information can be downloaded at https://www.mdpi.com/article/10.3390/nano14100851/s1: Figure S1. (a) Optical microscope image of the MoS_2_/WSe_2_/MoS_2_ BJT. (b–d) Time domain characteristics of the device operating at 1 Hz, 100 Hz, and 1 kHz, respectively. (e) The frequency response of the device. (f) The phase response of the device.

## References

[1] Liu Y., Weiss N.O., Duan X.D., Cheng H.C., Huang Y., Duan X.F. (2016). Van der Waals heterostructures and devices. Nat. Rev. Mater..

[2] Frisenda R., Molina-Mendoza A.J., Mueller T., Castellanos-Gomez A., Van D. (2018). Atomically thin p–n junctions based on two-dimensional materials. Chem. Soc. Rev..

[3] Castellanos-Gomez A., Duan X.F., Fei Z., Gutierrez H.R., Huang Y., Huang X.Y., Quereda J., Qian Q., Sutter E., Sutter P. (2022). Van der Waals heterostructures. Nat. Rev. Methods Primers.

[4] Park J.H., Park J.C., Yun S.J., Kim H., Luong D.H., Kim S.M., Choi S.H., Yang W., Kong J., Kim K.K. (2014). Large-Area Monolayer Hexagonal Boron Nitride on Pt Foil. ACS Nano.

[5] Ismach A., Chou H., Ferrer D.A., Wu Y.P., McDonnell S., Floresca H.C., Covacevich A., Pope C., Piner R., Kim M.J. (2012). Toward the Controlled Synthesis of Hexagonal Boron Nitride Films. ACS Nano.

[6] Li H., Yin Z.Y., He Q.Y., Li H., Huang X., Lu G., Fam D.W.H., Tok A.I.Y., Zhang Q., Zhang H. (2012). Fabrication of Single- and Multilayer MoS_2_ Film-Based Field-Effect Transistors for Sensing NO at Room Temperature. Small.

[7] Rhyee J.S., Kwon J., Dak P., Kim J.H., Kim S.M., Park J., Hong Y.K., Song W.G., Omkaram I., Alam M.A. (2016). High-Mobility Transistors Based on Large-Area and Highly Crystalline CVD-Grown MoSe_2_ Films on Insulating Substrates. Adv. Mater..

[8] Campbell P.M., Tarasov A., Joiner C.A., Tsai M.Y., Pavlidis G., Graham S., Ready W.J., Vogel E.M. (2016). Field-effect transistors based on wafer-scale, highly uniform few-layer p-type WSe_2_. Nanoscale.

[9] Zhou J.D., Lin J.H., Huang X.W., Zhou Y., Chen Y., Xia J., Wang H., Xie Y., Yu H.M., Lei J.C. (2018). A library of atomically thin metal chalcogenides. Nature.

[10] Novoselov K.S., Geim A.K., Morozov S.V., Jiang D., Zhang Y., Dubonos S.V., Grigorieva I.V., Firsov A.A. (2004). Electric field effect in atomically thin carbon films. Science.

[11] Guo W., Wu B., Wang S., Liu Y.Q. (2018). Controlling Fundamental Fluctuations for Reproducible Growth of Large Single-Crystal Graphene. ACS Nano.

[12] Guo W., Jing F., Xiao J., Zhou C., Lin Y.W., Wang S. (2016). Oxidative-Etching-Assisted Synthesis of Centimeter-Sized Single-Crystalline Graphene. Adv. Mater..

[13] Aftab S., Eom J. (2019). Van der Waals 2D layered-material bipolar transistor. 2D Mater..

[14] Su B.W., Zhang X.L., Yao B.W., Guo H.W., Li D.K., Chen X.D., Liu Z.B., Tian J.G. (2020). Laser Writable Multifunctional van der Waals Heterostructures. Small.

[15] Lee G., Pearton S.J., Ren F., Kim J. (2018). Two-Dimensionally Layered p-Black Phosphorus/n-MoS_2_/p-Black Phosphorus Heterojunctions. ACS Appl. Mater. Interfaces.

[16] Su B.W., Yao B.W., Zhang X.L., Huang K.X., Li D.K., Guo H.W., Li X.K., Chen X.D., Liu Z.B., Tian J.G. (2020). A gate-tunable symmetric bipolar junction transistor fabricated via femtosecond laser processing. Nanoscale Adv..

[17] Yang S.J., Pi L.J., Li L., Liu K.L., Pei K., Han W., Wang F.K., Zhuge F.W., Li H.Q., Cheng G. (2021). 2D Cu_9_S_5_/PtS_2_/WSe_2_ Double Heterojunction Bipolar Transistor with High Current Gain. Adv. Mater..

[18] Lee G., Pearton S.J., Ren F., Kim J. (2019). 2D Material-Based Vertical Double Heterojunction Bipolar Transistors with High Current Amplification. Adv. Electron. Mater..

[19] Liu L.W., Xu N.S., Zhang Y., Zhao P., Chen H.J., Deng S.Z. (2019). Van der Waals Bipolar Junction Transistor Using Vertically Stacked Two-Dimensional Atomic Crystals. Adv. Funct. Mater..

[20] Lin C.Y., Zhu X.D., Tsai S.H., Tsai S.P., Lei S.D., Shi Y.M., Li L.J., Huang S.J., Wu W.F., Yeh W.K. (2017). Atomic-Monolayer Two-Dimensional Lateral Quasi-Heterojunction Bipolar Transistors with Resonant Tunneling Phenomenon. ACS Nano.

[21] Li H., Ye L., Xu J.B. (2017). High-Performance Broadband Floating-Base Bipolar Phototransistor Based on WSe_2_/BP/MoS_2_ Heterostructure. ACS Photonics.

[22] Lv L., Zhuge F.W., Xie F.J., Xiong X.J., Zhang Q.F., Zhang N., Huang Y., Zhai T.Y. (2019). Reconfigurable two-dimensional optoelectronic devices enabled by local ferroelectric polarization. Nat. Commun..

[23] Afzal A.M., Iqbal M.Z., Dastgeer G., Nazir G., Mumtaz S., Usman M., Eom J. (2020). WS_2_/GeSe/WS_2_ Bipolar Transistor-Based Chemical Sensor with Fast Response and Recovery Times. ACS Appl. Mater. Interfaces.

[24] Liu L.W., Xu N.S., Ke Y.L., Chen H.J., Zhang Y., Deng S.Z. (2021). Sensing by Surface Work Function Modulation: High Performance Gas Sensing using van der Waals Stacked Bipolar Junction Transistor. Sens. Actuators B-Chem..

[25] Dastgeer G., Shahzad Z.M., Chae H., Kim Y.H., Ko B.M., Eom J. (2022). Bipolar Junction Transistor Exhibiting Excellent Output Characteristics with a Prompt Response against the Selective Protein. Adv. Funct. Mater..

[26] Yan Z., Xu N., Deng S. (2024). Realization of High Current Gain for Van der Waals MoS_2_/WSe_2_/MoS_2_ Bipolar Junction Transistor. Nanomaterials.

[27] Abraham M., Mohney S.E. (2017). Annealed Ag contacts to MoS_2_ field-effect transistors. J. Appl. Phys..

[28] Jiang Y.R., Wang R.Q., Li X.P., Ma Z.N., Li L., Su J., Yan Y., Song X.H., Xia C.X. (2021). Photovoltaic Field-Effect Photodiodes Based on Double van der Waals Heterojunctions. ACS Nano.

[29] Thakur D., Sato Y., Sabarigresan M., Ramadurai R., Balakrishnan V. (2022). Enhanced optical emission at MoS_2_-WS_2_ heterostructure interface with n-N junction. Appl. Surf. Sci..

[30] Xiao J.W., Zhang Y., Chen H.J., Xu N.S., Deng S.Z. (2018). Enhanced Performance of a Monolayer MoS_2_/WSe_2_ Heterojunction as a Photoelectrochemical Cathode. Nano-Micro Lett..

[31] Zhu J.Q., Yue X.F., Chen J.J., Wang J., Wan J., Bao W.Z., Hu L.G., Liu R., Cong C.X., Qiu Z.J. (2023). Ultrasensitive Phototransistor Based on Laser-Induced P-Type Doped WSe_2_/MoS_2_ Van der Waals Heterojunction. Appl. Sci..

