# Supplementary material for: AC Characteristics of van der Waals Bipolar Junction Transistors Using an MoS2/WSe2/MoS2 Heterostructure
Source: Nanomaterials (Basel). 2024 May 14;14(10):851. doi: 10.3390/nano14100851 (PMC11123697; doi:10.3390/nano14100851)
Supplement: Supplementary file 1 [file nanomaterials-14-00851-s001.zip › nanomaterials-2955079-supplementary.pdf]

nanomaterials

*Supplementary Material*

# AC Characteristics of van der Waals Bipolar Junction Transistors Using an $MoS_2/WSe_2/MoS_2$ Heterostructure

**Zezhang Yan, Ningsheng Xu and Shaozhi Deng ***

State Key Laboratory of Optoelectronic Materials and Technologies, Guangdong Province Key Laboratory of Display Material and Technology, School of Electronics and Information Technology, Sun Yat-Sen University, Guangzhou 510275, China; yanzzh3@mail2.sysu.edu.cn (Z.Y.); stsxns@mail.sysu.edu.cn (N.X.)

* Correspondence: stsdsz@mail.sysu.edu.cn

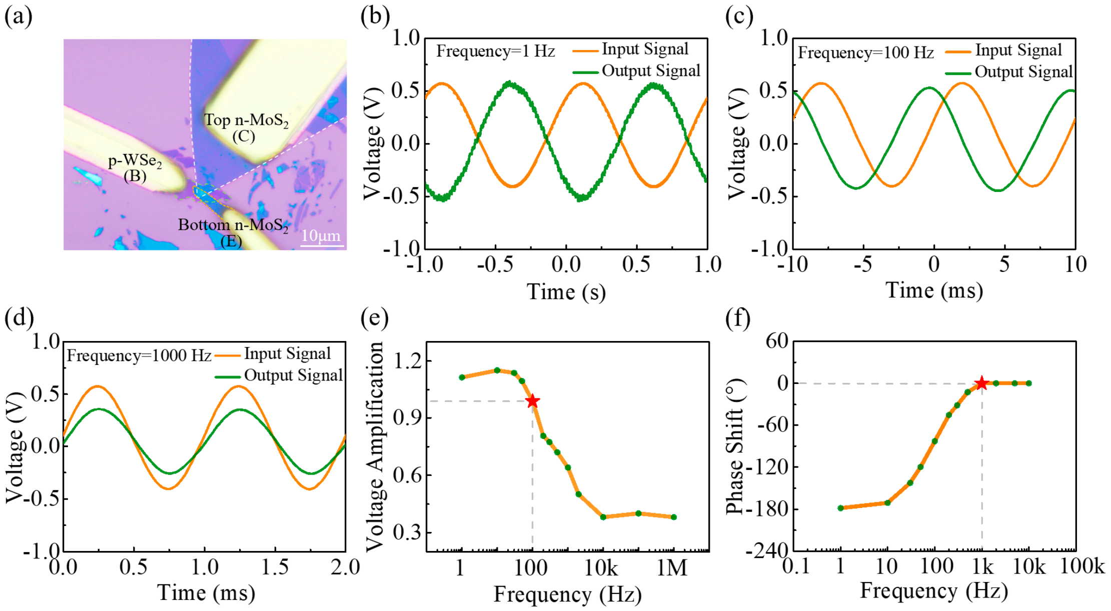


**Figure S1.** (a) Optical microscope image of the $MoS_2/WSe_2/MoS_2$ BJT, (b)-(d) Time-domain characteristics of the device operating at 1 Hz, 100 Hz, and 1 kHz, respectively, (e)The frequency response of the device, (f)The phase response of the device.
